# Analysis of player speed and angle toward the ball in soccer

**DOI:** 10.1038/s41598-024-62480-7

**Published:** 2024-05-23

**Authors:** Álvaro Novillo, Antonio Cordón-Carmona, Abraham García-Aliaga, Ignacio Refoyo Roman, Roberto López del Campo, Ricardo Resta, Javier M. Buldú

**Affiliations:** 1 Complex Systems Group, Universidad Rey Juan Carlos, C/ Tulipán, 28933 Móstoles, Madrid, Spain; 2 Grupo Interdisciplinar de Sistemas Complejos (GISC), 28933 Móstoles, Madrid, Spain; 3 Facultad de Ciencias de la Actividad Física y del Deporte (INEF-Departamento de Deportes), Universidad Politécnica de Madrid, C/Martín Fierro, 7, 28040 Madrid, Spain; 4 Mediacoach, LaLiga, Madrid, Spain

**Keywords:** Soccer, Match analysis, Performance, Tracking datasets, Player speed, Player behaviour, Physics, Scientific data, Statistics

## Abstract

The study analyzes how the magnitude and angle of the speed of soccer players change according to the distance to the ball and the phases of the game, namely the defensive and attacking phases. We observed how the role played in the team (goalkeeper, defender, midfielder, or forward) strongly determines the speed pattern of players. As a general trend, the speed’s modulus is incremented as their position is closer to the ball, however, it is slightly decreased when arriving at it. Next, we studied how the angle of the speed with the direction to the ball is related to the distance to the ball and the game phases. We observed that, during the defensive phase, goalkeepers are the players that run more parallel to the ball, while forwards are the ones running more directly to the ball position. Importantly, this behavior changes dramatically during the attacking phase. Finally, we show how the proposed methodology can be used to analyze the speed-angle patterns of specific players to understand better how they move on the pitch according to the distance to the ball.

## Introduction

Professional soccer is a sport of shared space and simultaneous participation, which increases the complexity level due to the interaction established between teammates and opponents. Under this framework, emergent and self-organized behaviors arise, leading to a complex interplay between the player movement and the player performance^1–5^. Rules are a fundamental part of every sport,for example, the offside rule in soccer restricts the player’s movements (i.e., location, speed, and acceleration) by reducing the space where the game is played^6^. As a result, teams must synchronize every action that takes place in a match, both in the offensive and defensive phases^7–9^, always with the target of scoring more goals than the opponent.

Previous research on speed analysis in soccer has shown that game speed increased on average from approximately 8.0–9.2 m/s over the period 1966–2010, i.e., an increase of 15%^10^, and game speed is predicted to continue increasing^11^. Interestingly, there is no difference in game speed between home and away games, but there is a difference in game speed between leagues^12^. When attackers move faster, defenders must adjust to this situation by performing different types of defense depending on their position on the pitch^13^, the place where the ball is (or could be) stolen^14,15^, or the players being either in front of or behind the ball^16^. Along these lines, some researchers have found that teams run longer distances and do so at a higher speed in the defensive phase than in the attacking phase^17,18^^,^^19^.

During a soccer match, running is performed at different speeds. High-intensity running is related to the success of a team, since teams at higher positions in the final ranking have players who perform more actions of this type^17^. This fact may be because the best teams aim to have greater initiative in the game, which can be achieved through faster movements of their players. However, Del Coso et al.^20^ found no association between the maximum running speed of teams and team position in the rankings at the end of a season. In addition to their importance in performance, high-speed sprints are also a common mechanism of injury with negative implications (i.e., financial, rehabilitation periods, risk of reinjury)^21^.

On the other hand, sprinting at maximum speed (> 24 km/h) occurs a smaller number of times than any other running intensity, with a distance covered of fewer meters per match^17^^,^^22^. However, it is worth noting that goals are preceded mainly by sprinting^23–25^. At the same time, a player’s running speed varies depending on the time of the match at which such running occurs, with 20% less distance covered in the last 15 min of a match compared to the first 15 min^3^. Finally, footballers have developed a faster sprint over time^26^ while running more meters at higher intensities^27^, differentiating between running with or without an opponent^24^ and running more often when winning than when drawing or losing^28^.

Even though the speed is a vector composed of both magnitude and direction, the latter has been analyzed to a lesser extent. In this regard, running in a straight direction is the most frequent goal^24,29^,however, diagonal running has also been shown to improve performance^30^. Concerning the position of players on the pitch, attackers perform more linear movements (towards the opponent’s goal), while defenders perform more lateral movements, intending to avoid the spaces that may have been generated in the defensive line^25,31^^,^^19^ For their part, the midfielders fluctuate between the two previous running styles, seeking to balance the team^31–33^.

In prior scientific investigations focused on indoor football, comprehensive analysis was conducted to scrutinize the angular disparities between the offensive and defensive phases of the game^34^. This research unveiled insights into the tactical dynamics of the soccer. The defensive team’s strategic positioning was observed to prioritize safeguarding critical spatial regions by effectively tracking and adapting to the positions of the ball and the defended goal, primarily through lateral movements. Conversely, the offensive team’s tactical objectives primarily revolved around disrupting the defensive team’s synchronization, primarily through longitudinal movements. These findings complement and align with the conclusions reached by previous analysis^35,36^, who delved into the aspects of player interactions with the ball in both offensive and defensive contexts. These works underscore the significant influence exerted by variables such as the ball’s trajectory, the goal’s spatial orientation, and the overarching objectives of the game itself in shaping the behavioral patterns exhibited by players on the indoor football field.

Within this framework, we analyzed players’ speed vectors (direction and magnitude) in the offensive and defensive phases, depending on their position, field zone, and distance to the ball. The main objective was to understand the speed patterns of players based on their proximity to the ball, differentiating between the roles they play on their team. To do this, we distinguished between 4 fundamental positions: goalkeeper, defender, midfielder, and forwards. Next, we used player tracking datasets to determine, at each moment, the players’ speed and distance to the ball, as well as the angle formed by the players’ running direction with the line connecting them to the ball. Then, we identified the field regions where the players were located and averaged the speeds based on the distance to the ball, field position, and the player’s role in the team. As we will see, the results show different speed patterns for players depending on their role in the team, the phase of play (defensive/offensive), and their position on the pitch.

## Materials and Methods

### Datasets

The goal of this research is to examine the relationship between a player’s velocity and their distance to the ball, as well as the angle formed between (i) their velocity vector and (ii) the vector from their current position to the ball’s position at any given moment in a game. To achieve this, tracking data from the Mediacoach® system was processed using an ensemble of statistical methods. Mediacoach® relies on the on-the-pitch acquisition of the player’s position using the Tracab Optical Tracking system, which is a multi-camera system that records each player’s position on the pitch at 25 frames per second. The multi-camera setup has three units, each with a resolution of 1920 × 1080 pixels. It provides a panoramic picture, creating a stereoscopic view for triangulating the players and the ball. The multi-camera system recovers the body’s skeleton, and then the center of the body is projected into the ground. In the event of a temporal loss of any location, an experienced operator corrects the position of the players. The datasets obtained by the Mediacoach® system have previously been validated with GPS^37,38^. Note that the correlation between the GPS measurements and the optical tracking depend on different variables, such as the speed and player position. However, as shown by Felipe et al.^37^ the agreement is very high in all cases. In this paper, we have used datasets acquired with the fourth generation of the Tracab Optical Tracking system. Concerning the accuracy of the reconstructed position of the players into a two-dimensional Euclidean space, Linke et al.^39^ have shown that this particular system has an accuracy of 9 cm (root mean square error, RMSE) when compared to a VICON motion capture system used as a reference^40^. With regard to the speed, the accuracy was 0.09 m/s (RMSE), being more accurate in the detection of high-speeds^39^.

The tracking data used in this study was obtained from the Spanish National League during the 2018/2019 season. To obtain interpretable results, we checked the two halves of each game and only considered player data for those games where such players had played at least a complete half. Note that including substitutes (or players who have been a few minutes on the pitch) could distort the results since they would be less fatigated than the rest. Following these restrictions, we analyzed 100 matches and obtained the position of 438 different players with a temporal resolution of 2 frames per second. The matches correspond to the first 10 fixtures of the season, comprising 10 matches for each team. This was achieved by extracting a frame every half-second, resulting in a total of 1,251,934 frames available for analysis. The dataset was downsampled to reduce computational demand. Together with the tracking datasets we also extracted the player position from LaLiga datasets. Player roles were categorized into four different positions: goalkeepers, defenders, midfielders, and forwards. We only considered players who played for more than 45 min in a match. Under this condition we have analyzed a total of 438 performances, which according to the position occupied by players can be divided into 28 goalkeepers, 149 defenders, 169 midfielders and 92 forwards’ performances. We obtained the approval from the Ethics Committee of the Polytechnical University of Madrid (Madrid, Spain) to carry out this research (project FDRED00000-DML-DATOS-20230609) and publish the results in an open access journal.

### Methodology and statistical analysis

**Figure 1:**
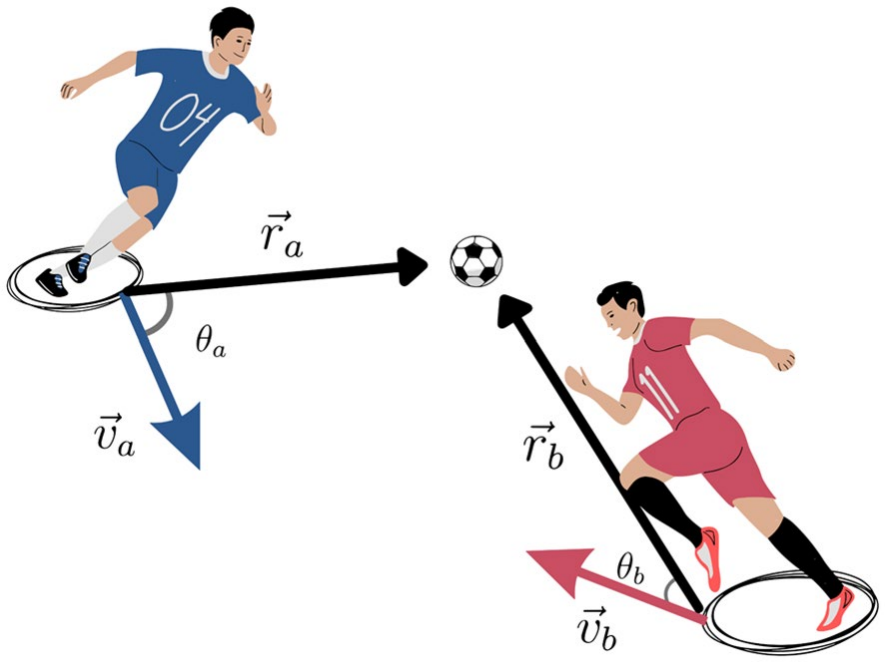
Qualitative description of the player’s speed and angle. The distance to the ball of player $i$ is the modulus of $\overrightarrow{{r}_{i}}$, and the angle ${\theta }_{i}$ is computed using the scalar product between $\overrightarrow{{r}_{i}}$ and $\overrightarrow{{v}_{i}}$ . In the figure, players $a$ and $b$ run at different speeds, $\overrightarrow{{v}_{a}}$ and $\overrightarrow{{v}_{b}}$, and different angles, ${\theta }_{a}$, and ${\theta }_{b}$, respectively.

To study the players’ behavior with respect to the ball, we obtained (i) the distance vector between each player and the ball $\overrightarrow{{r}_{i}}$ at each frame of the match and (ii) the angle ${\theta }_{i}$ between the player’s speed vector $\overrightarrow{{v}_{i}}$ and the distance vector $\overrightarrow{{r}_{i}}$ (See Fig. 1 for details). The analysis was conducted using two different approaches: a continuous approach, where all velocities and angles were computed inside a circle with an integer radius R ⊂ [0,50] m, and a discretized approach, where we distinguished between 3 different regions based on the distance between the player and the ball: short distance [0, 3) m, medium distance [3, 10) m, and long distance [10, + inf) m.

**Figure 2:**
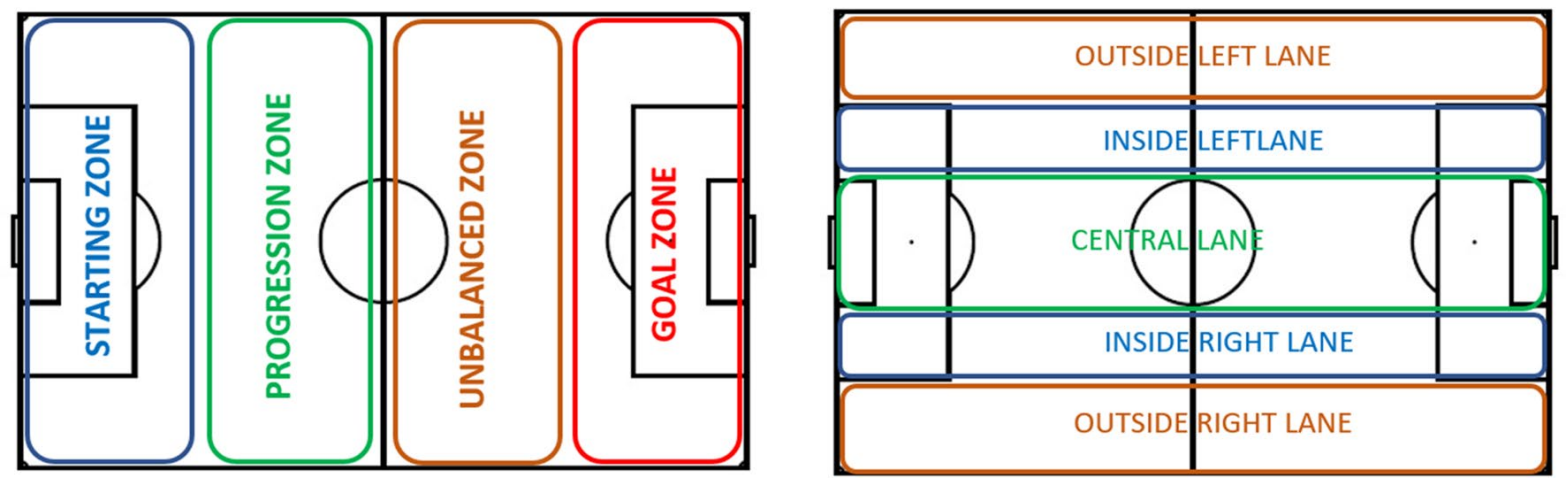
Division of the pitch. We considered 2 divisions of the pitch, one regarding the opponent’s goal proximity (left plot) and another with regard to the side lanes (right plot). The former is divided into four regions, while the latter is divided into five regions, each related to a tactical conception of the game. Finally, both divisions are joined into a single one, leading to 4 × 5 pitch divisions (i.e., containing 20 different pitch regions).

The study also considered the players’ location on the pitch, specifically looking at the players’ speed in relation to their distance from the ball at the different areas of the pitch (See Fig. 2 for details).

Different statistical visualizations, such as campograms, scatter plots, or box-plots, were used to analyze the relationship between the players’ distance to the ball and their velocity. The pitch regions and players’ velocities were combined to create a spatial distribution of the players’ positions in a 4 × 5 spatial grid. The time that each player stayed in each zone of the grid was used to characterize their speed pattern throughout the matches. This particular pitch partition is based on soccer coaching nomenclature, with modifications from Fernández-Navarro et al.^41^. Concerning the analysis of the different variable (speeds, angles and distances), it includes two kinds of statistics: the arithmetic mean and the probability distribution function. The equation for calculating the arithmetic mean $\overline{{\varvec{x}} }$ of a set of observations ${x}_{i}$ is:

1 $$ \overline{x} = \frac{1}{n}\left( {\mathop \sum \limits_{i = 1}^{n} x_{i} } \right) = \frac{{x_{1} + x_{2} + x_{3} + \cdots + x_{n} }}{n} $$

where $n$ is the total number of observations. Concerning the probability $P\left({x}_{i}\right)$ of observing a value ${x}_{i}$, it is obtained as:

2 $$ P\left( {x_{i} } \right) = \frac{{m_{i} }}{M} $$

where ${m}_{i}$ accounts for the number of times that an observation ${x}_{i}$ belongs to the bin ${B}_{j}$. The length ${L}_{B}$ of the bin ${B}_{j}$ is obtained by discretizing the interval given by ${x}_{max}-{x}_{min}$. In all cases, we divided the interval into $S=50$ partitions, obtaining a set of $S$ consecutive bins of length ${L}_{B} =({x}_{max}-{x}_{\text{min}})/50$.

## Results

**Figure 3:**
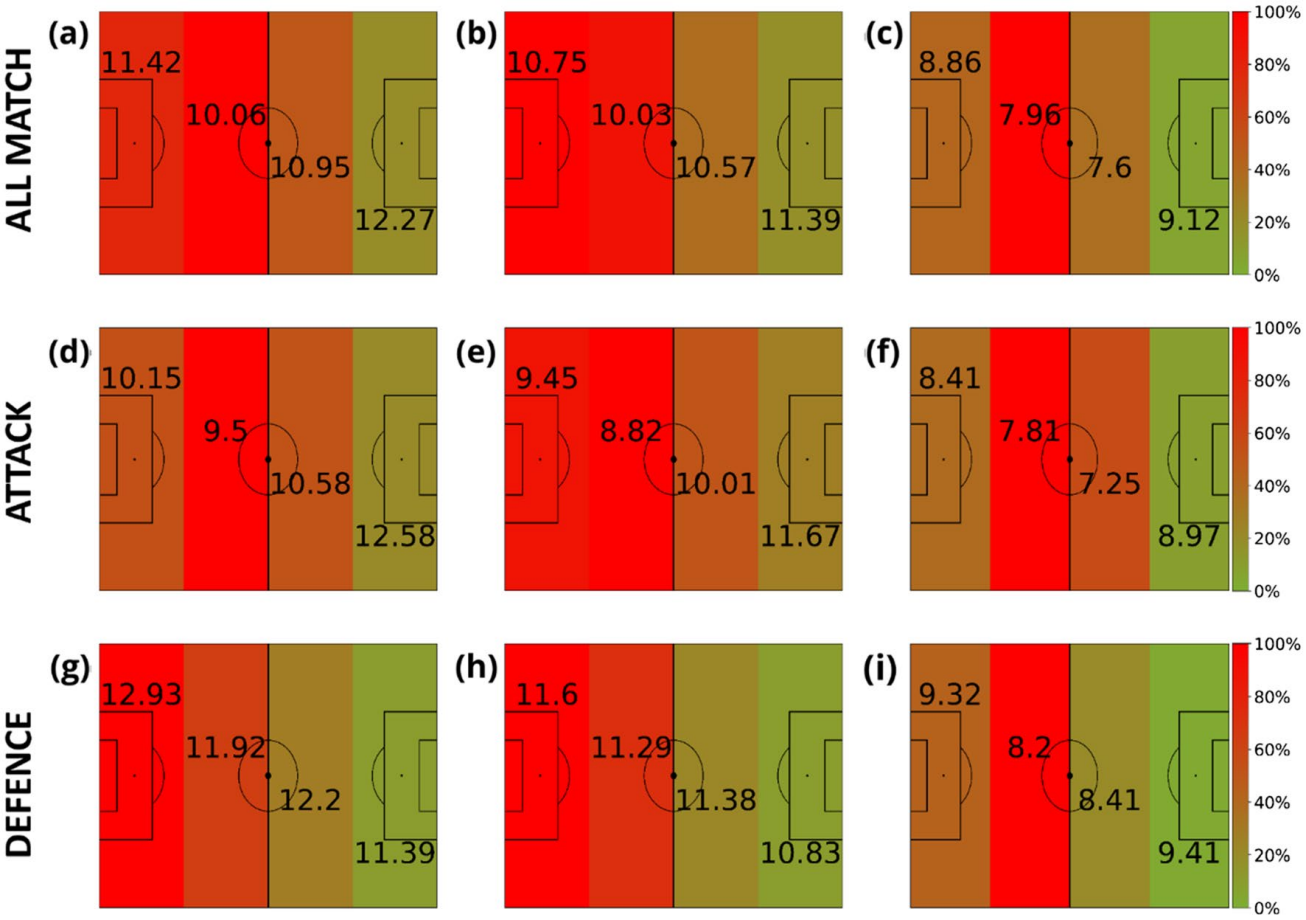
Average velocities (km/h) of players in terms of their distance to the ball. The intervals of the distance to the ball were: [0, 3) meters (first column), [3, 10) meters (second column) and (10, inf) meters (last column). Note that the values displayed in each zone are the average velocity of players in that region of the field, and the heatmap colors represent the amount of time these players spent inside each region. This value is normalized by the maximum amount of time obtained at each plot. Each row represents different game phases: the first row contains all phases (attacking and defending phases combined), the second corresponds to the attacking phase, and the third is the defending phase. The divisions are the ones proposed in Fig. 2.

Our analysis starts by measuring the speed of all players without paying attention to their role in the team. Figure 3 shows the average speed of players according to a division of the pitch regarding the proximity to the opponent’s goal. The analysis is carried out for the three different distance windows: (A-D-G) short distance [0, 3) m, (B-E-H) medium distance [3, 10) m and (C-F-I) long distance [10, + inf) m. The figure’s colors are not related to the speed but to the time spent at each region. The first row of Fig. 3 is obtained for all phases of the game. We can see how the closer to the ball (Fig. 3A), the faster players move. Furthermore, the campograms allow us to see how the speed is increased when players are closer to the goals, both their own and the opponent’s one. Since the direction of attack is from left to right, we can observe how players spend less time closer to the opponent’s goal. Finally, the second and third rows of Fig. 3 show the same results but splitting the game into offensive (middle row) and defensive (bottom row) phases. Comparing both figures, we can observe how the speed is always higher during the defensive phase.

**Figure 4:**
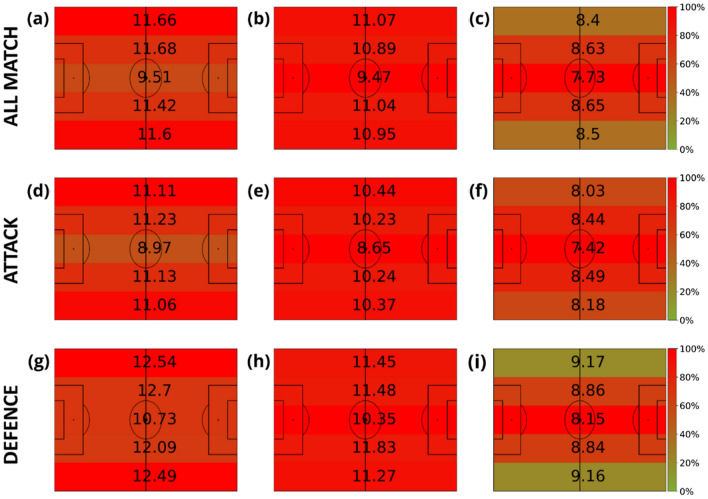
Average velocities (km/h) of players in terms of their distance to the ball. The intervals of the distance to the ball were: [0, 3) meters (first column), [3, 10) meters (second column) and (10, inf) meters (last column). Note that the values displayed in each zone are the average velocity of all the players in that region of the field, and the heatmap colors represent the amount of time these players spent inside each region. This value is normalized by the maximum amount of time obtained at each plot. Each row represents different game phases: the first row contains all phases (attacking and defending phases combined), the second corresponds to the attacking phase, and the third is the defending phase. The divisions are the ones proposed in Fig. 2.

A similar analysis has been conducted with a division of the pitch into side lanes. Figure 4 shows how players move faster when running close to the most lateral lanes and also that the highest speeds are always detected when players are closer to the ball.

**Figure 5:**
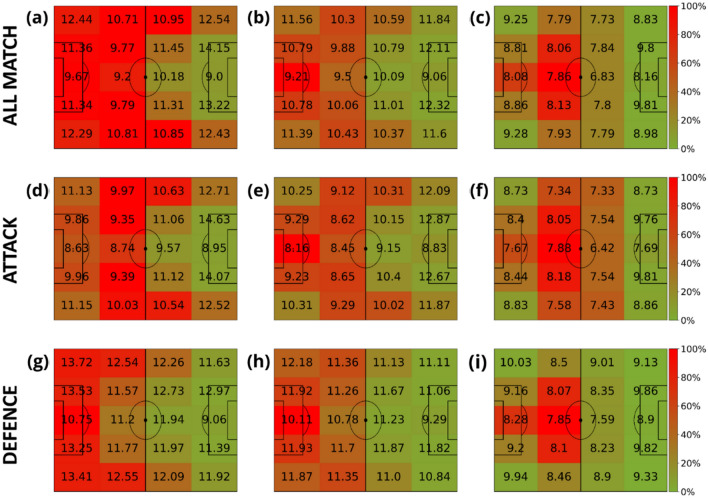
Average velocities (km/h) of players in terms of their distance to the ball. The intervals of the distance to the ball were: [0, 3) meters (first column), [3, 10) meters (second column) and (10, inf) meters (last column). Note that the values displayed in each zone are the average velocity of players in that region of the field, and the heatmap colors represent the amount of time these players spent inside each region. This value is normalized by the maximum amount of time obtained at each plot. Each row represents different game phases: the first row contains all phases (attacking and defending phases combined), the second corresponds to the attacking phase, and the third is the defending phase. In this case, the spatial partition combines the ones proposed at Figs. 3 and 4, leading to a 4 × 5 grid.

Figure 5 shows the results of combining both kinds of pitch divisions. The 4 × 5 spatial partition gives us more insights into the spatial distribution of the velocities with respect to the distance to the ball. This extended partition highlights that players run faster when: (i) close to the ball, (ii) close to the four corners of the pitch, and (iii) when entering the opponent’s box on both sides.

**Figure 6:**
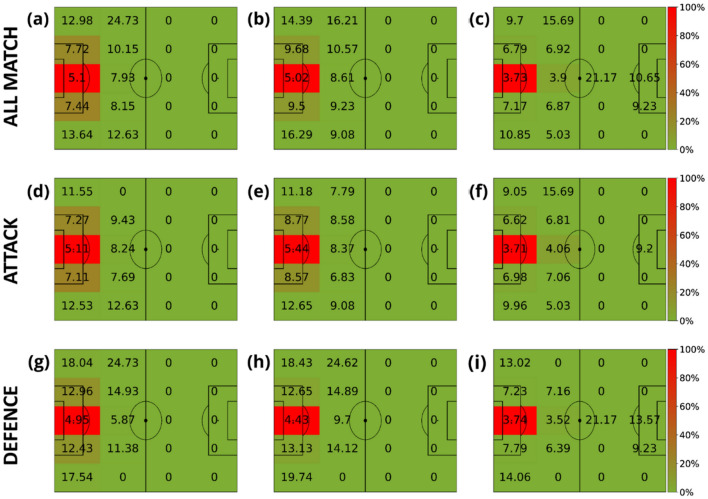
Average velocities (km/h) of goalkeepers in terms of their distance to the ball. The intervals of the distance to the ball were: [0, 3) meters (first column), [3, 10) meters (second column) and (10, inf) meters (last column). The heatmap colors represent the normalized amount of time these players spent inside each region. Each row represents different phases of the game: the first row contains all phases, the second corresponds to the attacking phase, and the third is the defending phase.

**Figure 7:**
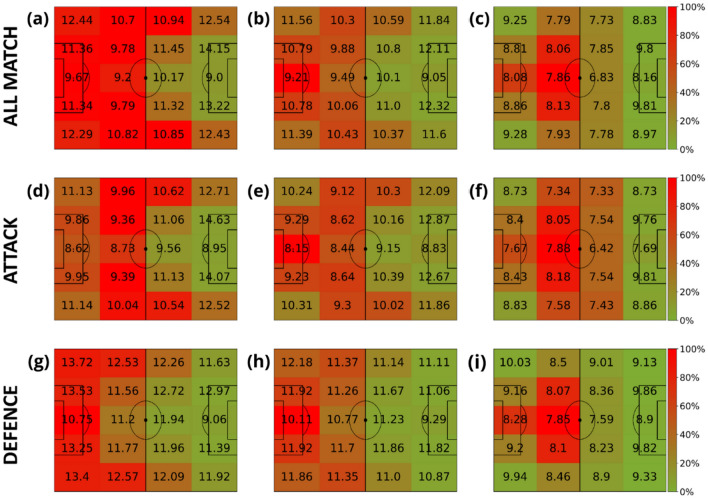
Average velocities (km/h) of defenders in terms of their distance to the ball. The intervals of the distance to the ball were: [0, 3) meters (first column), [3, 10) meters (second column) and (10, inf) meters (last column). The heatmap colors represent the normalized amount of time these players spent inside each region. Each row represents different game phases: the first row contains all phases, the second corresponds to the attacking phase, and the third is the defending phase.

**Figure 8:**
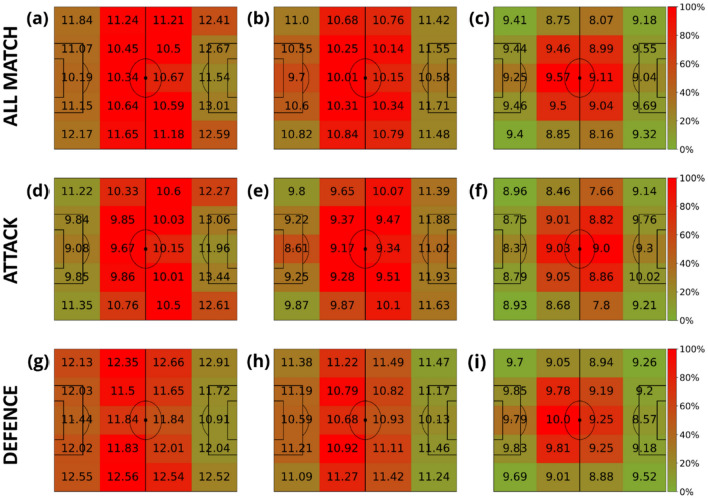
Average velocities (km/h) of midfielders in terms of their distance to the ball. The intervals of the distance to the ball were: [0, 3) meters (first column), [3, 10) meters (second column) and (10, inf) meters (last column). The heatmap colors represent the normalized amount of time these players spent inside each region. Each row represents different game phases: the first row contains all phases, the second corresponds to the attacking phase, and the third is the defending phase.

**Figure 9:**
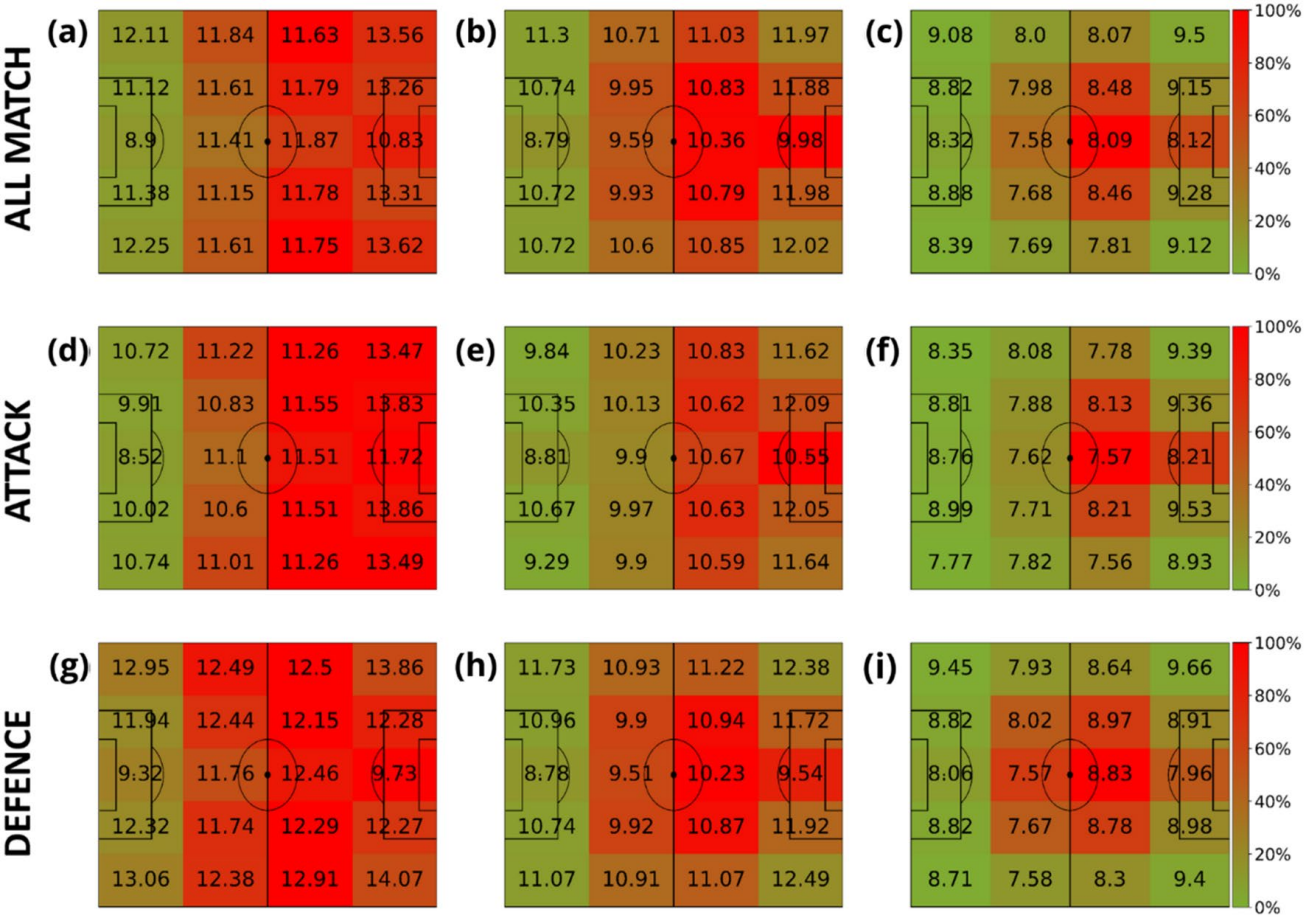
Average velocities (km/h) of forwards in terms of their distance to the ball. The intervals of the distance to the ball were: [0, 3) meters (first column), [3, 10) meters (second column) and (10, inf) meters (last column). The heatmap colors represent the normalized amount of time these players spent inside each region. Each row represents different game phases: the first row contains all phases, the second corresponds to the attacking phase, and the third is the defending phase.

The next step is to study the spatial distribution of the speed for each role a player has in the team. Specifically, we defined four main roles: goalkeepers, defenders, midfielders, and forwards. Figures 6, 7, 8 and 9 contain the average speeds of players according to their specific role and their position on the pitch. Note how the role of the player is related to the time spent at each specific region. We can observe how, except for the goalkeeper, speeds tend to be higher when players run closer to the ball.

**Figure 10:**
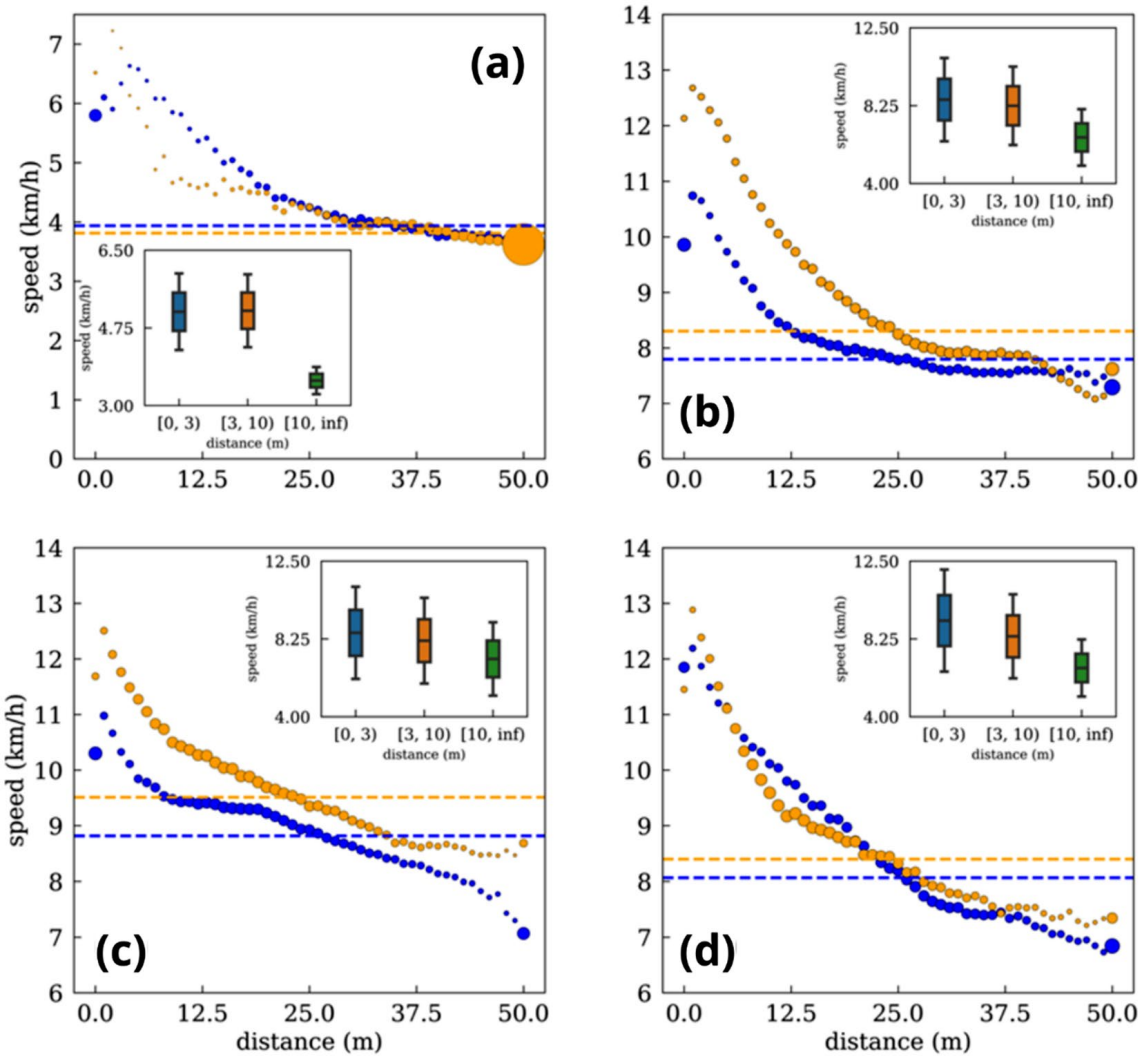
Average speed at different distances from the ball. (**a**) Goalkeepers. (**b**) Defenders. (**c**) Midfielders. (**d**) Forwards. Orange circles indicate the speed averages while defending, and blue points are the speed averages while attacking. The dashed orange line represents the average defending speed, and the blue one is the average attacking speed. The inset shows the speed distributions in the three discrete intervals. The size of the circles for each position is proportional to the time spent at each distance [see Fig. 11 for a more detailed analysis].

Once we have characterized the general scenario in which players run faster when closer to the ball, we extend this analysis further considering a continuous interval of distances to the ball. In this way, we obtain players’ speed for any distance R ⊂ [0,50] m, with a difference of 1 m between two consecutive points. Figure 10 shows the smooth decay of the speeds’ modulus as players get farther from the ball. Additionally, we have split the analysis between the attacking and defensive phases. We can observe how, for defenders, midfielders, and forwards, the speed is higher during the defensive phase. This difference is especially higher, in the first two cases, at close distances to the ball. Interestingly, forward players display very similar velocity patterns at the attacking and defensive phases at all ranges of distances. Contrary to these cases, goalkeepers’ average speed is very similar in both phases while displaying different patterns at distances closer to the ball.

Importantly, in all positions, there is a general behavior where the average speed decreases when the player is within a distance of approximately 2 m from the ball. This phenomenon can be explained by the need for players to reduce their velocity to control the ball.

**Figure 11:**
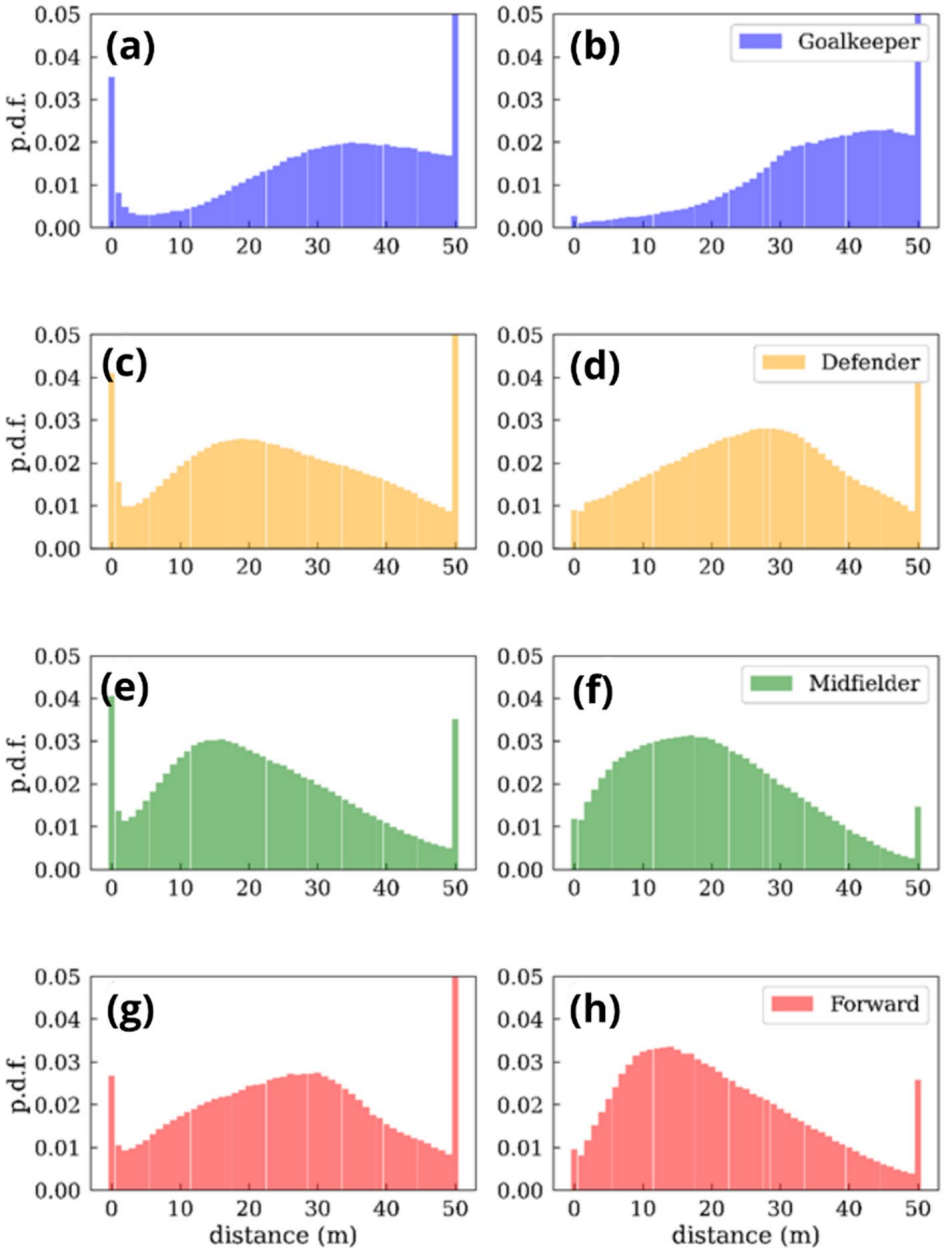
Time spent by players at different distances to the ball. For each player role, we plot the probability distribution function (p.d.f.) of the time spent at each distance to the ball. Attacking (**a**, **c**, **e** and **g**) and defending (**b**, **d**, **f** and **h**) phases.

However, the previous results must be interpreted with regard to the time each player has spent at different distances from the ball. Figure 11 shows the probability distribution function of the time spent at each distance for the four different roles assigned to players. Generally, we can observe a maximum located at intermediate distances, with the goalkeepers having a peak farther away. However, two significant maxima appear for distances close to 1 m and larger than 50 m. The latter, which appears both at the attacking and defending phases, can be explained by the fact that we are considering a broader region of parameters (any R > 50 m) instead of narrow windows of 1 m, as is the case of the rest of the bars displayed in Fig. 11. On the other hand, the increase of the time reported for distances closer to 1 m can be explained by a tendency of players of coming closer to the ball when controlling it since this peak only appears during the attacking phase (Fig. 11a).

**Figure 12:**
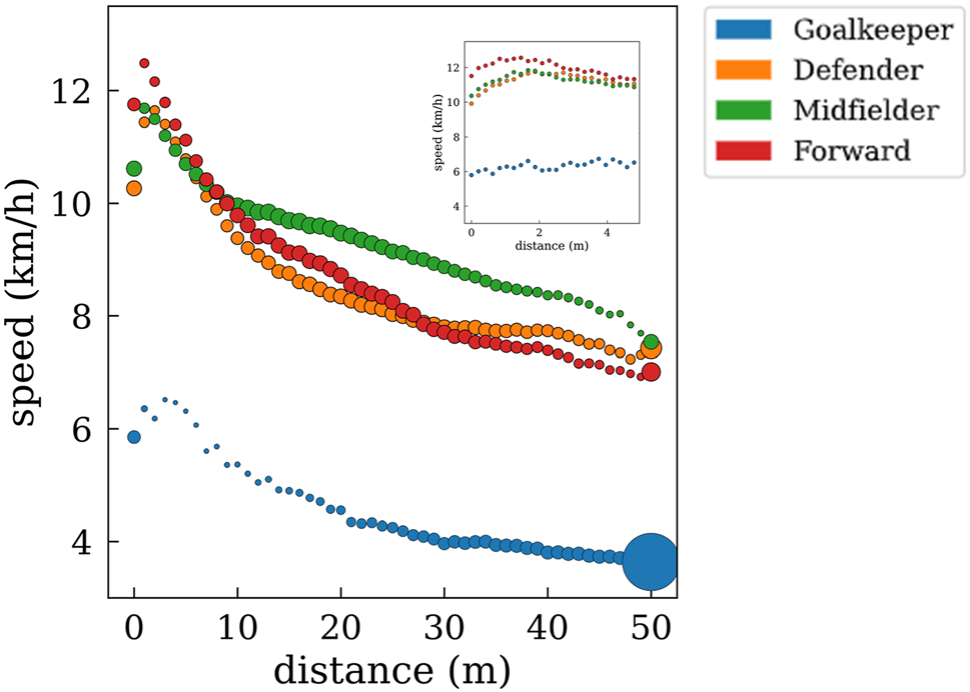
Scatter plot of the average speed as a function of the distance to the ball *at all game phases.* Goalkeepers (blue), defenders (orange), midfielders (green), and forwards (red). The size of the points is proportional to the p.d.f of the time spent at each distance.

**Figure 13:**
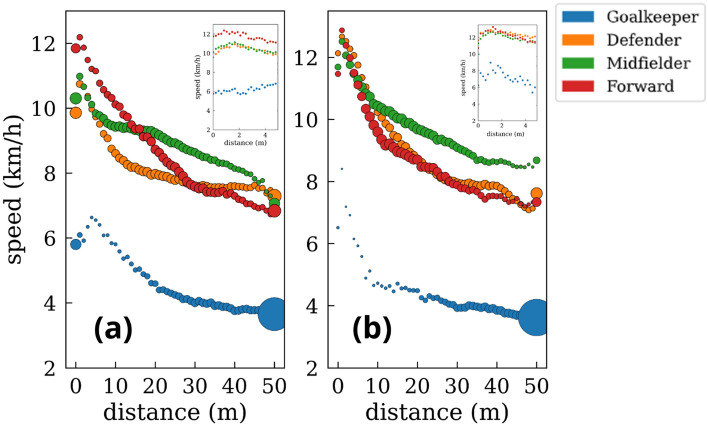
Differences in the average speed of the players as a function of the distance to the ball for the (**a**) attacking and (**b**) defensive phases*.* Goalkeepers (blue), defenders (orange), midfielders (green), and forwards (red). The size of the points is proportional to the p.d.f of the time spent at each distance.

Figure 12 shows a comparison of the speed-distance plot for the four defined roles of a player. We can observe that forwards are the fastest players when they are closer to the ball, but midfielders have the highest average speeds at longer distances from the ball. The same comparison is shown in Fig. 13, but, in this case, we have split the defensive and offensive phases. Interestingly, despite being the fastest players when closer to the ball, this behavior is only reported during the attacking phase.

**Figure 14:**
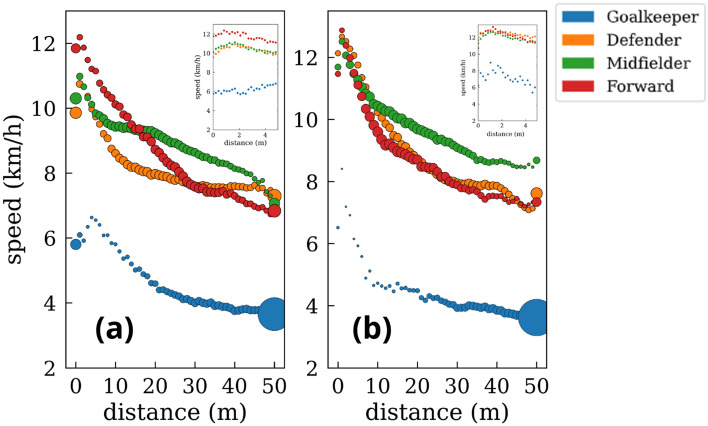
Scatter plot of the average angle between the velocity of the players and their distance to the ball vector (see Fig. 1 for details). Goalkeepers (**a**), defenders (**b**), midfielders (**c**), and forwards (**d**). Blue and orange colors correspond to the attacking and defending phases, respectively. The dashed orange line represents the average angle while defending, while the blue one is the average attacking angle. The size of the points is proportional to the p.d.f of the time spent at each distance.

**Figure 15:**
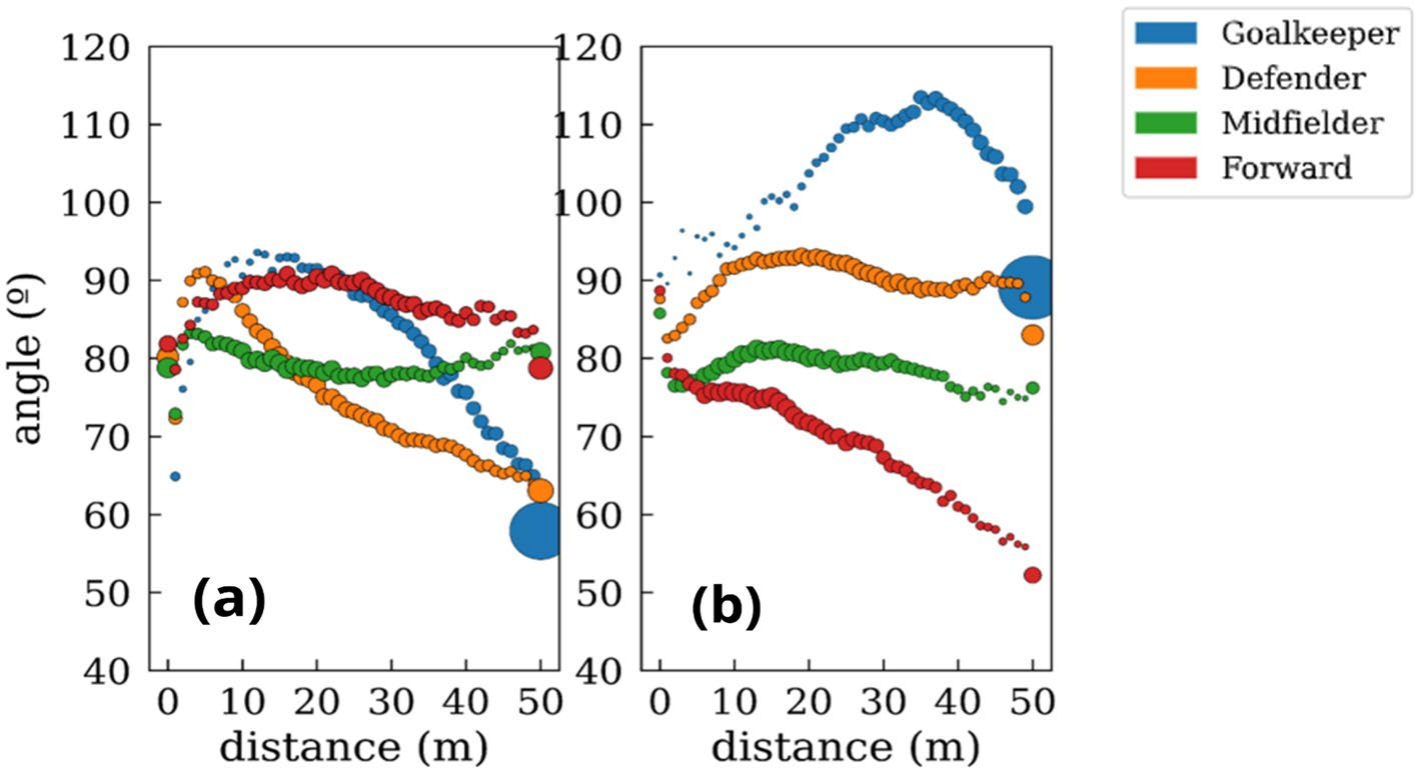
Scatter plot of the average angle between the velocity of the players and their distance to the ball vector at (**a**) attacking and (**b**) defending phases*.* Goalkeepers (blue), defenders (orange), midfielders (green), and forwards (red). The size of the points for each position is proportional to the p.d.f of the time spent at each distance.

If we consider that speed is a vector (i.e., not a scalar magnitude), we can go one step beyond its module and pay attention to the direction toward players are running. As explained in the Methods section, we can calculate the angle that the speed of a player has with respect to the ball. In this way, we can measure when a player is running directly toward the ball (angle close to 0 degrees) or parallel to it (angle close to 90 degrees). Figure 14 shows how the angle towards the ball changes according to the distance from the ball and the role of each player. Goalkeepers (Fig. 3A) are the players with a higher angle at any distance. This fact indicates that goalkeepers are prone to move parallel to the ball and not toward it. This behavior is enhanced during the defensive phase and can be explained since goalkeepers maintain their position close to their goal. On the other hand, it is worth noting the differences in the angle towards the ball for the three roles of the field players. Defenders move more parallel to the ball when defending and increase the probability of moving towards it when attacking (Fig. 3B). However, this behavior changes when they are very close (R < 5 m) to the ball. Forwards behave oppositely and run more frequently towards the ball when defending (Fig. 3D). Finally, midfielders (Fig. 3C) move in a very similar when way when attacking and defending, a situation that it is in the middle of the defender and forward behavior. Figure 15 shows a comparison between the player positions split in the attacking and defensive phases. In the Supplementary Material, we also included the speed of the players in the analysis. In Fig. S1, we measured the angle of players without considering the phase of the match, but including a color code that indicates the speed of players. In this way, we can observe the relation of the speed, angle, and distance to the ball. In Table 1, we summarized the average values of the speed modulus and the angle towards the ball of all cases studied.

**Table 1:** Average speeds and angles towards the ball for each position of players and phases of the game.

	Average speed $(\text{km}/\text{h})$			Average angle (°)		
	Total	Attacking	Defensive	Total	Attacking	Defensive
Goalkeepers	$3.873\pm 0.003$	$3.937\pm 0.004$	$3.812\pm 0.004$	$92.399\pm 0.863$	$82.576\pm 1.339$	$103.314\pm 1.026$
Defenders	$8.057\pm 0.003$	$7.798\pm 0.004$	$8.304\pm 0.004$	$82.648\pm 0.592$	$75.068\pm 1.180$	$89.821\pm 0.378$
Midfielders	$9.161\pm 0.003$	$8.817\pm 0.004$	$9.511\pm 0.004$	$78.961\pm 0.138$	$79.392\pm 0.264$	$78.492\pm 0.308$
Forwards	$8.222\pm 0.004$	$8.064\pm 0.006$	$8.399\pm 0.006$	$78.857\pm 0.337$	$87.050\pm 0.000$	$68.695\pm 1.080$

Finally, the Supplementary Material contains a scatter plot of the average angles between the velocity and the distance to the ball (Fig. S1). Figure S2 shows some examples of the speed-distance patterns of different players of the Spanish national league. We included the average pattern of the speed-distance for each player role (goalkeeper, defender, midfielder and forward) to compare the player performance with their colleagues at the same position. In Fig. S3, we show the angle-distance patterns for the same players analyzed in Fig. S2. Finally, Fig. S4 contains the campograms of the players analyzed in Fig. S3. Due to the number of figures generated, we will only show the 4 × 5 discretized campogram for each player, where the figure layout is the same as the one displayed in Figs. 5, 6, 7, 8 and 9.

## Discussion

This research aimed to analyze the relationship between a soccer player’s velocity and the distance to the ball, as well as the angle formed between the velocity vector and the vector towards the ball. The analysis was conducted treating the player speed in two different ways: (i) averaging player speed into three categories according to the distance to the ball (up to 3 m, from 3 to 10 m, more that 10 m far from the ball) and (ii) considering the average speed at any distance ranging from 0.5 to 50 m to the ball. While the second methodology is more accurate, there is an agreement in the conclusions of both kind of analysis despite the averaging into 3 regions simplifies the analysis and assumes a subjective partition of the data. Similar to previous observations ^19^^,^^17,18^, we found that players run at a higher speed during the defensive phase, taking into account the average speed across the entire soccer field, except for goalkeepers, who run slightly faster during the attacking phase and when the ball is far away. From a methodological standpoint, the goalkeeper’s movement pattern may be attributed to the need to cover the open spaces between the defenders and the goal when they are far from the goal. This requires them to run longer distances. Furthermore, the new goal kick rule, which includes goalkeepers in the first line of the ball, makes them a part of the attacking formation. This can lead to an increase in their speed during attacking phases. Consequently, goalkeepers move almost as much as the outfield players and are often positioned between the two central defenders to facilitate ball possession during attacks and reduce the space between the lines.

We have also seen how players move faster when they run closer to the most lateral lanes. This result reinforces the findings of^19^, who showed that with ball possession, “running along the side lanes” is correlated with players running faster^17,42^. The explanation could be related to the fact that side lanes are the places of the pitch where there are more free spaces, compared to the central lanes. In addition, a common tactical resource is to locate faster players occupying these positions on the pitch with the aim of creating separation from the defensive players and providing crosses into the penalty area from the opposition and finishing areas^32^^,^^19^^,^^25^. Consequently, the areas comprising the four corners of the pitch and the areas entering the opponent’s box have the highest speeds, demonstrating that these are key areas for creating goal-scoring opportunities and advantageous one-on-one plays. Therefore, players on the side lanes seek to break the opponent’s defensive line with their speed, probably because in the central areas there is a greater density of players who are slower and more technical than their counterparts.

Players spend the most time in the progression zone and the central lanes, probably because they apply defensive pressure to quickly steal the ball and arrive faster to the opponent’s goal^14,15^. Interestingly, players exhibit higher speeds when they are near the ball^31,32^, either to pressure an opponent or to outrun their defender. These tactical concepts, which categorize players based on their proximity to the ball and opponents, explain how soccer, despite being a collective sport, requires individuals to effectively resolve group situations through quick thinking and decision-making. With this regard, sprinting to harass an opponent regardless of the role of the players (forward, midfielder, or defender) is crucial in current soccer teams^31,32^^,^^43^ , since 1-on-1 pressure is nowadays more extended than zonal pressure. As seen in Fig. 10, forwards are the players with the highest peak speed (at close distances to the ball), a fact that is more accentuated in the offensive phase, possibly due to the performance of clearance behind the defensive line (these being challenging to defend)^23–25^. At the same time, midfielders have the highest average speed (9.16 km/h). However, at distances greater than ~ 25 m, there are no significant differences in players’ velocity patterns during defensive and offensive phases for all positions^25,30–32^. On the other hand, goalkeeper and forward speeds show the least differences between offensive and defensive phases across the range of the distances studied. Concerning the forwards, this fact can be explained by the difficulty of receiving the ball in advanced areas and by the offside rule, leading on many occasions to the reception of the ball backward to the opponent’s goal, unlike defenders and midfielders, who usually receive the ball while facing the goal.

It is worth noting that, at distances less than 2 m from the ball, speed decreases, which may be due to the technical difficulty of controlling the ball^16^, ineffective dribbling, or the difficulty of overtaking the opponent with dribble^14,15^.

On the other hand, we also observed significant differences in the direction of the velocity of players depending on their position. Goalkeepers, who occupy a very specific position, are the players who have the greatest angle at any distance to the ball, indicating that their movements are parallel to the ball and not towards it. This behavior is accentuated during the defensive phase (103.31° on average) and can be explained by the fact that goalkeepers maintain their position close to the goal, where their high-intensity actions will be decisive in the match^44^. When comparing the angle towards the ball for the remaining three positions, we observed that defenders move more parallel to the ball when defending (89.82°) (they try to close the spaces between lines and get closer together) and are more likely to move towards the ball when attacking (75.07°). In contrast, forwards are more likely to run toward the ball when defending (68.69°) than attacking, moving more parallel to it (87.05). This fact is probably due to the defensive pressure in a 1-on-1 format, which is essential in the defensive phase as they are the first defenders of the team. Finally, we observed that midfielders move similarly when attacking (79.39°) and defending (78.49°).

In the individual player’s specific analysis (see Supplementary Materials), we observed that Player D’s running speed is below the average at almost any distance from the ball, and the angle to the ball is approximately 60°, i.e., not as the average of his position. If we analyze his movements on video, in the attacking phase, Player D usually performs a clearance by joining the players on the front line and then separating laterally to receive the ball in an oriented manner; from there, he either shoots or looks for a pass behind the line or to the wing to give continuity to the game. Additionally, our speed analysis may indicate that he keeps energy by exerting less physical effort in defense, allowing him to make higher-intensity efforts with less fatigue during the attacking phase. In contrast, Player B is a player who is quicker than average in his position and spends more time near the ball, and, on the other hand, Player C’s speed is about average in his position (defender) on the pitch. Finally, Player A has a pattern of angles in terms of distance that is less than average, which could be because he is a goalkeeper who provides support in the offensive phase of his team or because he provides covering behind the back of the defensive line.

Because of all, given the wide availability of tracking data in soccer and the possibilities for performance improvement, a deeper understanding of how soccer players move according to distance to the ball, their roles, and the regions of the pitch will allow coaches, analysts, physical trainers, etc., to improve both their game understanding and evaluation. As we have seen, both the modulus and the angle of the speed are key factors in the way players run and crucially depend on the distance to the ball and the phase of the game. We believe that further work taking into account the technical and tactical actions occurring at the same instant is necessary. For example, linking velocity data to the type of event in which they are obtained would provide a better understanding of soccer and the specificities of the actions that occur during a match. This fact is probably the main limitation of our study, since were unable to relate the match events (goals, shots, dribbles, …) in terms of the angles and speed of the runs. If we had been able to do so, we would have been able to identify the key moments where specific speed vectors were produced, such as during goal actions. Consequently, distinctions between forward, lateral, or backward displacements are not possible. We anticipate significant variations in body orientation relative to the ball and nearest opponent, which warrants further investigation. Additionally, if we can access skeleton reconstruction using pattern recognition algorithms, we could delve deeper into analyzing body balance and player speed. Hopefully, the development and availability of tracking datasets and the increasing computation capacity will result in further studies on player speed and, next, acceleration.

### Supplementary Information

Supplementary Figures.

## Data Availability

The data supporting the conclusions of this article will be made available by the authors under personal request.

## Code availability

The calculations have been carried out by an in-house software developed with Matlab. All codes are available to download through GitHub^22^.

## References

[1] Aguiar M, Gonçalves B, Botelho G, Lemmink K, Sampaio J (2015). Footballers’ movement behaviour during 2-, 3-, 4- and 5-a-side small-sided games. J. Sports Sci..

[2] Casamichana D, Castellano J, Calleja-Gonzalez J, San Román J, Castagna C (2013). Relationship between indicators of training load in soccer players. J. Strength Cond. Res..

[3] Duarte R, Araujo D, Correia V, Davids K (2012). Sports teams as superorganisms: Implications of sociobioloical models of behaviour for research and practice in team sports performance analysis. Sports Med..

[4] Duarte R, Araujo D, Folgado H, Esteves P, Marques P, Davids K (2013). Capturing complex, non-linear team behaviours during competitivesoccer performance. J. Syst. Sci. Complex..

[5] Ribeiro J, Silva P, Duarte R, Davids K, Garganta J (2017). Team sports performance analysed through the lens of social network theory: Implications for research and practice. Sports Med..

[6] IFAB. 2023. *RULE 11: OFFSIDE*, Accessed 9th October, 2023. [Online]: https://www.theifab.com/es/laws/latest/offside/#offside-position

[7] Folgado H, Duarte R, Marques P, Gonçalves B, Sampaio J (2018). Exploring how movement synchronization is related to match outcome in elite professional football. Sci. Med. Footb..

[8] Sampaio J, Maçãs V (2012). Measuring tactical behaviour in football. Int. J. Sports Med..

[9] Yamamoto Y, Yokoyama K (2011). Common and unique network dynamics in soccer games. PLoS ONE.

[10] Wallace JL, Norton KI (2014). Evolution of World Cup soccer final games 1966–2010: Game structure, speed and play patterns. J. Sci. Med. Sport.

[11] Nassis GP, Massey A, Jacobsen P, Brito J, Randers MB, Castagna C, Mohr M, Krustrup PJSJOM, Sports SI (2020). Elitesoccer of 2030 will not be the same as that of 2020: Preparing players, coaches, and support staff for the evolution. Scand. J. Med. Sci. Sports.

[12] Connor M, Mernagh D, Beato M (2022). Quantifying and modelling the game speed outputs of English Championship soccer players. Res. Sports Med..

[13] Fernandez-Navarro J, Ruiz-Ruiz C, Zubillaga A, Fradua L (2020). Tactical variables related to gaining the ball in advanced zones of the soccer pitch: Analysis of differences among elite teams and the effect of contextual variables. Front. Psychol..

[14] Lago-Ballesteros J, Lago-Peñas C, Rey E (2012). The effect of playing tactics and situational variables on achieving score-box possessions in a professional soccer team. J. Sports Sci..

[15] Vogelbein M, Nopp S, Hökelmann A (2014). Defensive transition in soccer—are prompt possession regains a measure of success? A quantitative analysis of German Fußball-Bundesliga 2010/2011. J. Sports Sci..

[16] Rein R, Raabe D, Memmert D (2017). “Which pass is better?” Novel approaches to assess passing effectiveness in elite soccer. Hum. Mov. Sci..

[17] Di Salvo V, Gegson W, Atkinson G, Tordoff P, Drust B (2009). Analysis of high intensity activity in premier league soccer. Int. J. Sports Med..

[18] Rampinini E, Impellizzeri FM, Castagna C, Coutts AJ, Wisløff U (2009). Technical performance during soccer matches of the Italian Serie A league: Effect of fatigue and competitive level. J. Sci. Med. Sport..

[19] Caldbeck P, Dos’santos T (2022). How do soccer players sprint from a tactical context? Observations of an English Premier League soccer team. J. Sports Sci..

[20] Del Coso J, Brito Souza D, Moreno-Perez V, Buldú JM, Nevado F, Resta R, López Del Campo R (2020). Influence of players’ maximum running speed on the team’s ranking position at the end of the Spanish LaLiga. Int. J. Environ. Res. Public Health.

[21] Schuermans J, Van Tiggelen D, Palmans T, Danneels L, Witvrouw E (2017). Deviating running kinematics and hamstring injury susceptibility in male soccer players: Cause or consequence?. Gait & Posture.

[22] Rey E, Kalén A, Lorenzo-Martínez M, López-Del Campo R, Nevado-Garrosa F, Lago-Peñas C (2024). elite soccer players do not cover less distance in the second half of the matches when game interruptions are considered. J. Strength Cond. Res..

[23] Cometti G, Maffiuletti NA, Pousson M, Chatard JC, Maffulli N (2001). Isokinetic strength and anaerobic power of elite, subelite and amateur French soccer players. Int. J. Sports Med..

[24] Faude O, Koch T, Meyer T (2012). Straight sprinting is the most frequent action in goal situations in professional football. J. Sports Sci..

[25] Martínez-Hernández D, Quinn M, Jones P (2023). Linear advancing actions followed by deceleration and turn are the most common movements preceding goals in male professional soccer. Sci. Med. Footb..

[26] Haugen TA, Tønnessen E, Hisdal J, Seiler S (2014). The role and development of sprinting speed in soccer. Int. J. Sports Physiol. Perform..

[27] Bush M, Barnes C, Archer DT, Hogg B, Bradley PS (2015). Evolution of match performance parameters for various playing positions in the English Premier League. Hum. Mov. Sci..

[28] Oliva-Lozano JM, Rojas-Valverde D, Gómez-Carmona CD, Fortes V, Pino-Ortega J (2020). Worst case scenario match analysis and contextual variables in professional soccer players: A longitudinal study. Biol. Sport.

[29] Sarmento H, Marcelino R, Anguera MT, Campaniço J, Matos N, Leitão JC (2014). Match analysis in football: A systematic review. J. Sports Sci..

[30] Cordón-Carmona A, García-Aliaga A, Marquina M, Calvo JL, Mon-López D, Refoyo Roman I (2020). What Is the relevance in the passing action between the passer and the receiver in soccer? Study of elite soccer in La Liga. Int. J. Environ. Res. Public Health.

[31] Ju W, Doran D, Hawkins R, Evans M, Laws A, Bradley P (2023). Contextualised high-intensity running profiles of elite soccer players with reference to general and specialised tactical roles. Biol. Sport.

[32] Ade J, Fitzpatrick J, Bradley PS (2016). High-intensity efforts in elite soccer matches and associated movement patterns, technical skills and tactical actions. Information for position-specific training drills. J. Sports Sci..

[33] Carling C, Le Gall F, Dupont G (2012). Analysis of repeated high-intensity running performance in professional soccer. J. Sports Sci..

[34] Travassos B, Araújo D, Duarte R, McGarry T (2012). Spatiotemporal coordination behaviors in futsal (indoor football) are guided by informational game constraints. Human Mov. Sci..

[35] Frencken, W. G. P., & Lemmink, K. A. P. M. (2008). Team kinematics of small-sided soccer games: A systematic approach. *Sci. Football VI*, 187–192.

[36] Travassos B, Araújo D, Vilar L, McGarry T (2011). Interpersonal coordination and ball dynamics in futsal (indoor football). Human Mov. Sci..

[37] Felipe JL, Garcia-Unanue J, Viejo-Romero D, Navandar A, Sánchez-Sánchez J (2019). Validation of a video-based performance analysis system (Mediacoach®) to analyze the physical demands during matches in LaLiga. Sensors.

[38] Pons E, García-Calvo T, Resta R, Blanco H, López Del Campo R, Díaz García J, Pulido JJ (2019). A comparison of a GPS device and a multi-camera video technology during official soccer matches: Agreement between systems. PLoS ONE.

[39] Linke D, Link D, Lames M (2020). Football-specific validity of TRACAB’s optical video tracking systems. PLoS ONE.

[40] Vicon, 2023. Accessed on 9th October, 2023, https://www.vicon.com/software/nexus/

[41] Fernandez-Navarro J, Fradua L, Zubillaga A, Ford PR, McRobert AP (2016). Attacking and defensive styles of play in soccer: Analysis of Spanish and English elite teams. J. Sports Sci..

[42] Bradley PS, Sheldon W, Wooster B, Olsen P, Boanas P, Krustrup P (2009). High-intensity running in English FA Premier League soccer matches. J. Sports Sci..

[43] Mcburnie AJ, Dos'santos T (2022). Multidirectional speed in youth soccer players: Theoretical underpinnings. Strength Cond. J..

[44] Di Salvo V, Benito PJ, Calderón FJ, Di Salvo M, Pigozzi F (2008). Activity profile of elite goalkeepers duringsoccer match-play. J. Sports Med. Phys. Fitness.

[45] GitHub, 2024. https://github.com/AlvaroNovillo/Analysis-of-player-speed-and-angle-toward-the-ball-in-soccer

[46] Harper DJ, Sandford GN, Clubb J, Young M, Taberner M, Rhodes D, Carling C, Kiely J (2021). Elitesoccer of 2030 will not be the same as that of 2020: What has evolved and what needs to evolve?. Scand. J. Med. Sci. Sports.

