# Author Correction: Analysis of player speed and angle toward the ball in soccer

**DOI:** 10.1038/s41598-024-64173-7

**Published:** 2024-06-07

**Authors:** Álvaro Novillo, Antonio Cordón-Carmona, Abraham García-Aliaga, Ignacio Refoyo Roman, Roberto López del Campo, Ricardo Resta, Javier M. Buldú

**Affiliations:** 1 Complex Systems Group, Universidad Rey Juan Carlos, C/ Tulipán, 28933 Móstoles, Madrid, Spain; 2 Grupo Interdisciplinar de Sistemas Complejos (GISC), 28933 Móstoles, Madrid, Spain; 3 Facultad de Ciencias de la Actividad Física y del Deporte (INEF-Departamento de Deportes), Universidad Politécnica de Madrid, C/Martín Fierro, 7, 28040 Madrid, Spain; 4 Mediacoach, LaLiga, Madrid, Spain

Correction to: *Scientific Reports* 10.1038/s41598-024-62480-7, published online 23 May 2024

The original version of this Article contained an error in the name of author Roberto López del Campo, which was incorrectly given as Robeto López del Campo.

The original Article has been corrected.

