# Supplementary material for: Analysis of player speed and angle toward the ball in soccer
Source: Sci Rep. 2024 May 23;14:11780. doi: 10.1038/s41598-024-62480-7 (PMC11116510; doi:10.1038/s41598-024-62480-7)
Supplement: Supplementary file 1 — Supplementary Figures. [file 41598_2024_62480_MOESM1_ESM.docx]

***Supplementary Material***

**Analysis of the player speed and angle towards the ball in soccer**

**Álvaro Novillo, Antonio Cordón-Carmona, Abraham García-Aliaga, Ignacio Refoyo Roman, R. López del Campo, R. Resta, and Javier M. Buldú**

1. **Supplementary Figures and Tables**
   1. **Supplementary Figures**

**
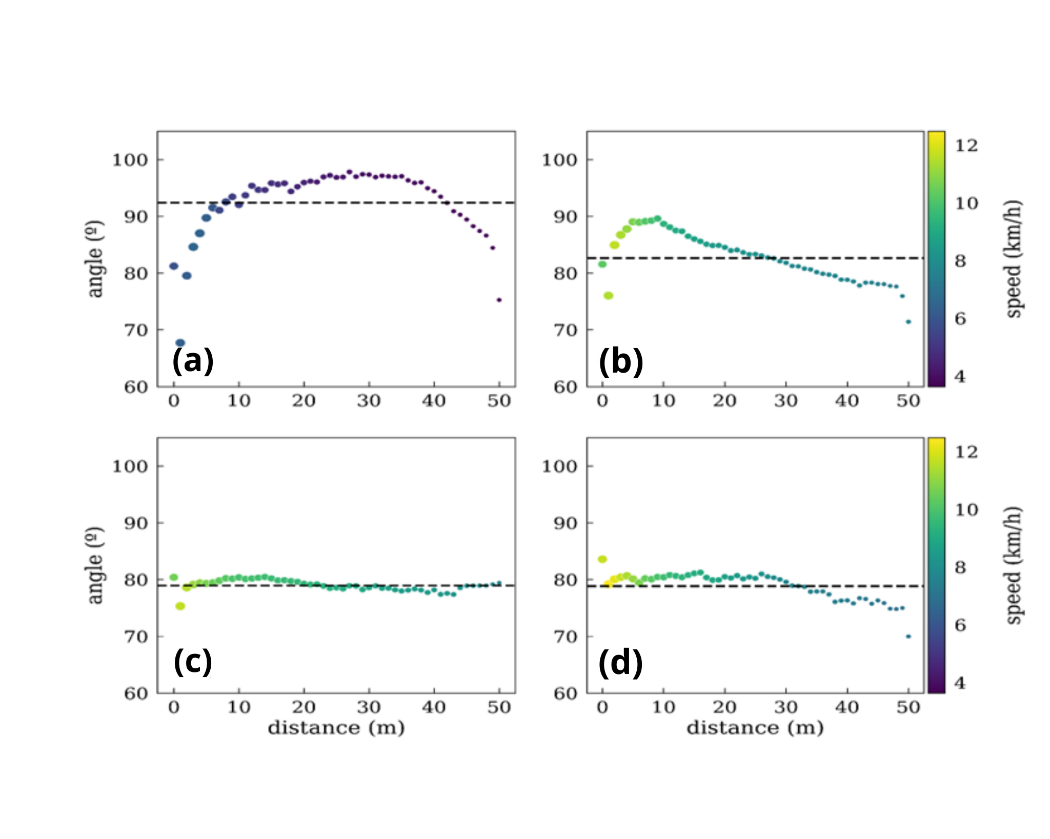
**

**Figure S1.** Scatter plot of the average angles between the velocity and the distance to the ball. (a) Goalkeepers, (b) defenders, (c) midfielders, and (d) forwards. The color gradient represents the players’ mean velocity at such distance to the ball (the lighter the color and the bigger the point, the faster the player moves).

In the following we show the particular performances of 4 different players of LaLiga. The four of them are top-leading players in their teams and positions. Player A is a goalkeeper, Player B is a midfielder, Player C is a defender and Player D is a forward.

**
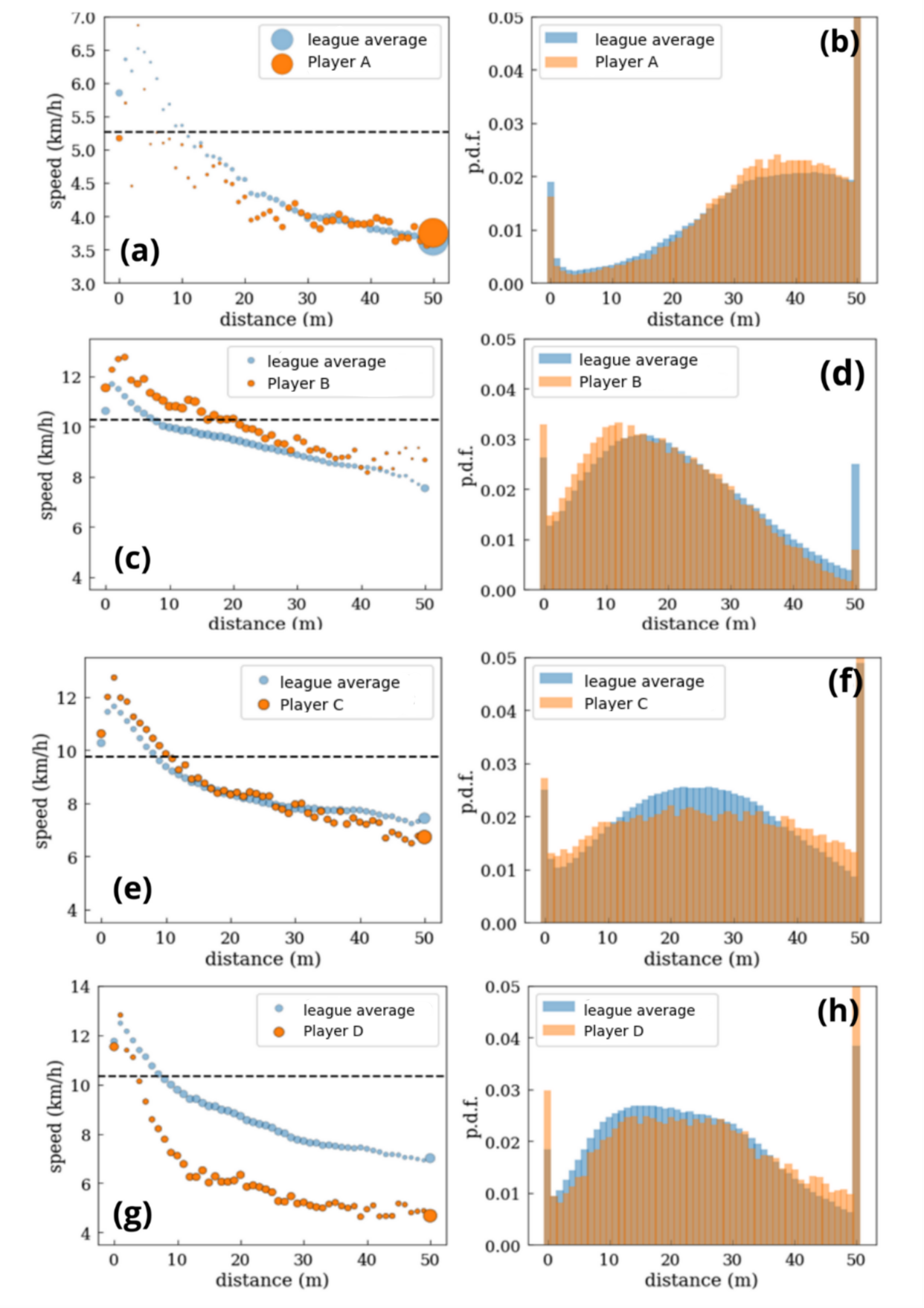
**

**Figure S2**. (a, c, e and g) Speed-distance performance of different players of LaLiga. Comparison between the league average speed (blue dots) with respect to each particular player. The dashed black line represents the average velocity of such position, for all distances to the ball. (b, d, f and h) p.d.f. of the time spent at each distance of the player (orange bars) with respect to the average (blue bars).

**
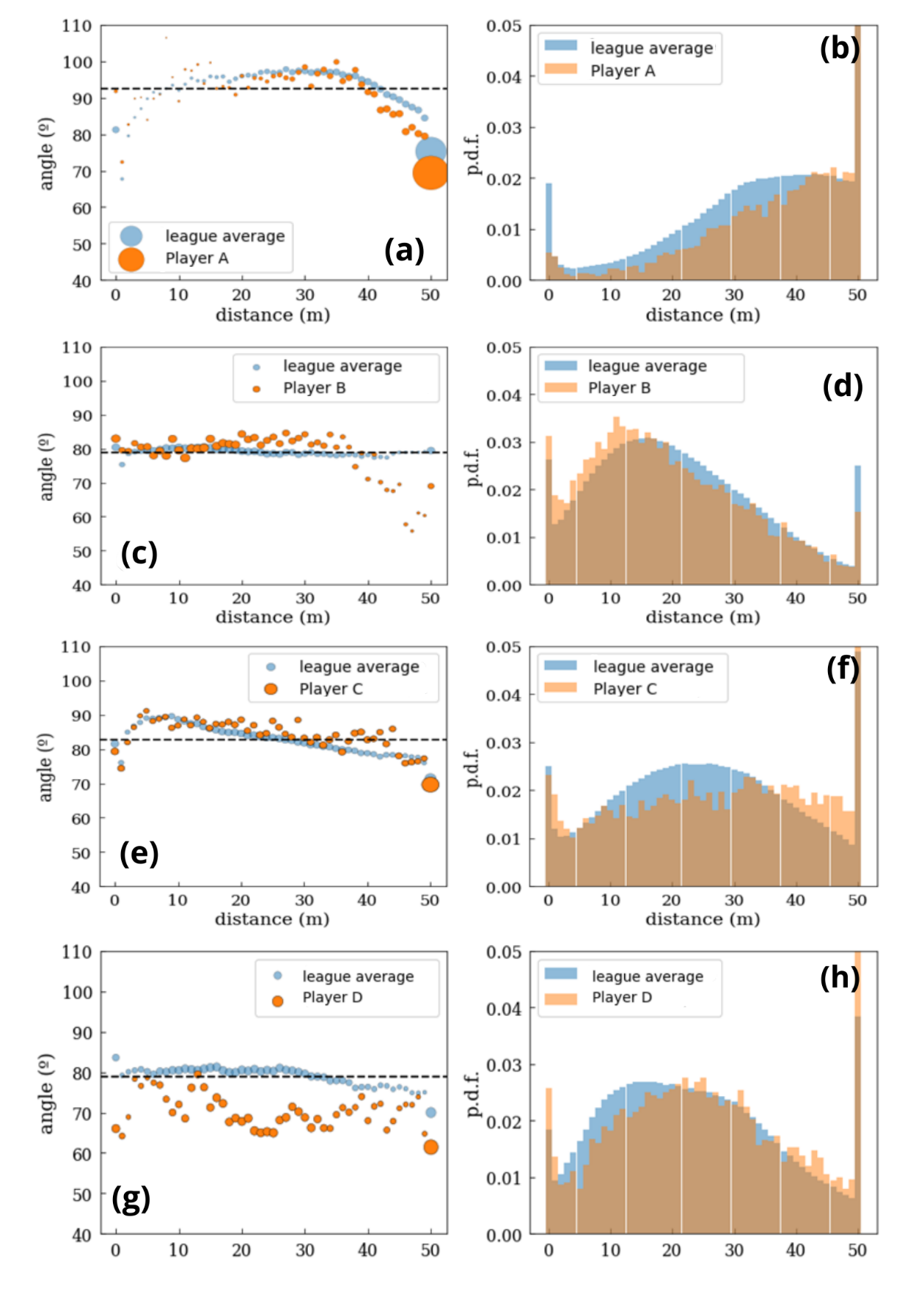
**

**Figure S3.** (a, c, e and g) Angle-distance performance of different players of LaLiga. Comparison between the league average angle-distance performance and different players. The black dashed line corresponds to the average angle for all distances. (b, d, f and h) p.d.f. of the time spent at each distance of the player (orange bars) with respect to the average (blue bars).


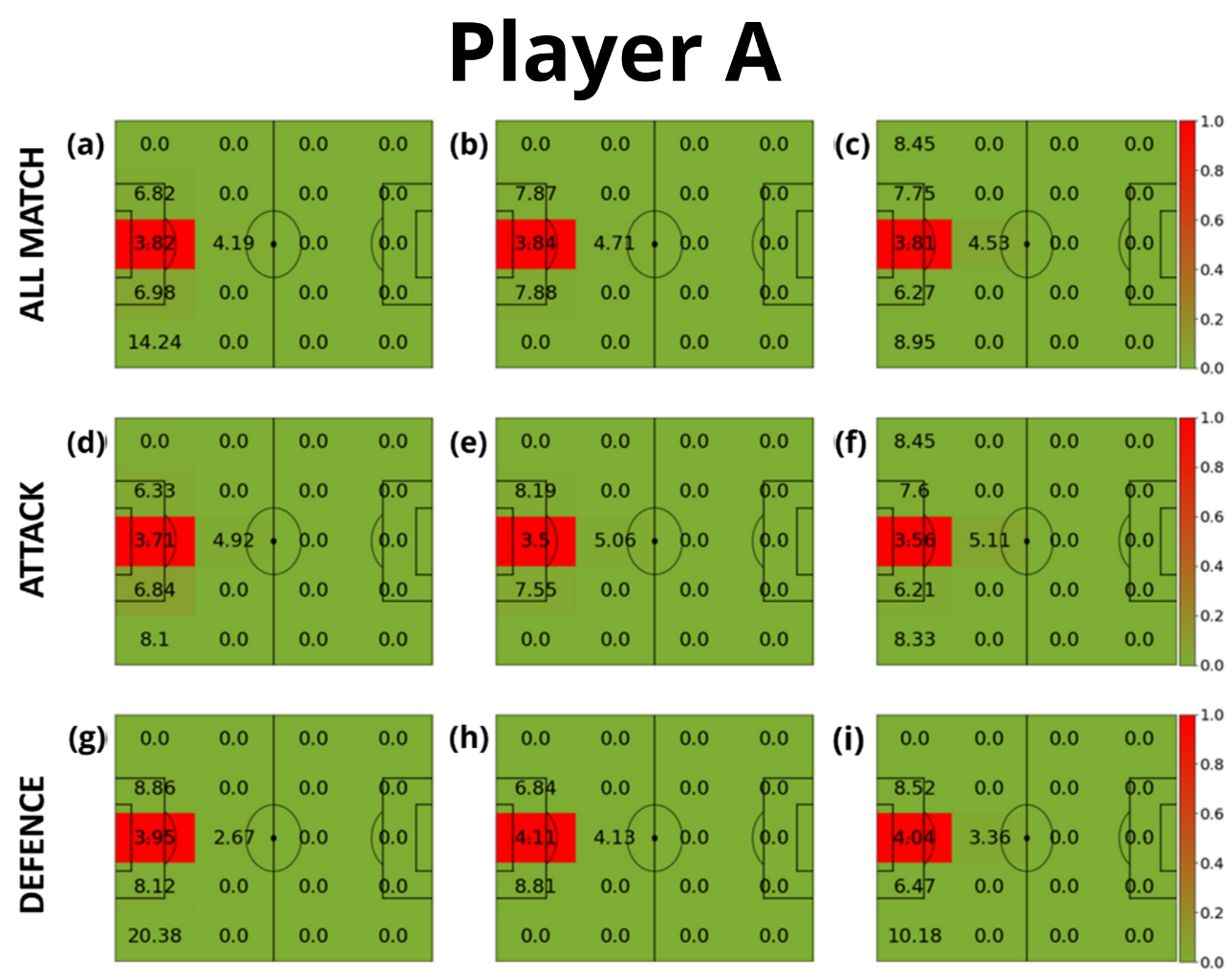


**Figure S4.** Mean velocity (in km/h) of Player A (goalkeeper) in terms of his distance to the ball. The intervals of the distance to the ball were: [0, 3) meters (first column), [3, 10) meters (second column) and (10, inf) meters (last column). Note that the values displayed in each zone is the average velocity of the player in that region of the field, and the heatmap represents the average amount of time this player has spent inside that region. This value is normalized by the maximum amount of time expended by the player in a specific region. Each row represents different instances of the game: the first row are all instances of the game (attacking and defending phases combined), the second is the attacking phase, and the third is the defending phase. In this case, the spatial partition is a combination of the ones proposed in Figs. 1,2, creating a 5x4 grid.


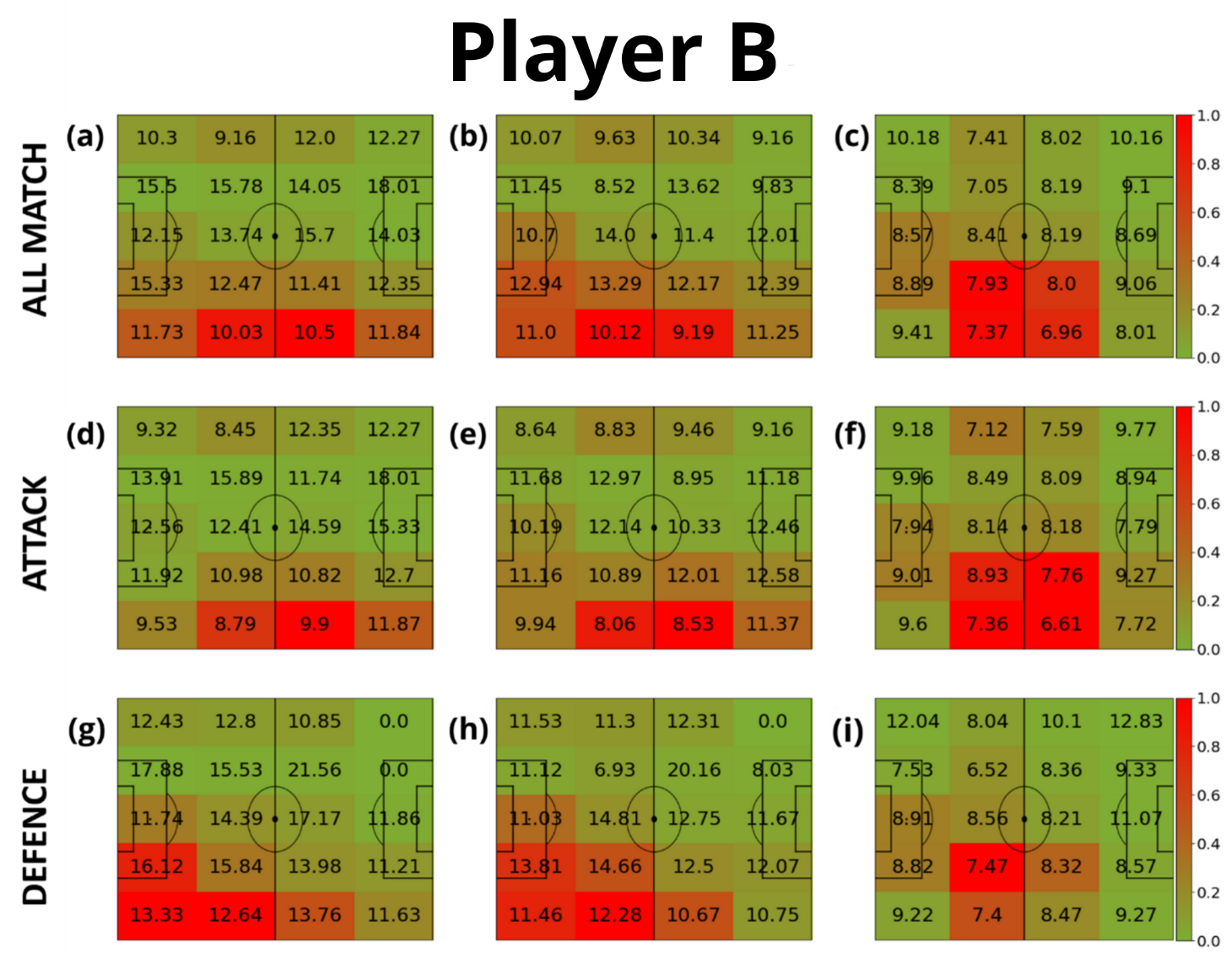


**Figure S5.** Mean velocity (in km/h) of Player B (defender) in terms of his distance to the ball. The intervals of the distance to the ball were: [0, 3) meters (first column), [3, 10) meters (second column) and (10, inf) meters (last column). Note that the values displayed in each zone is the average velocity of the player in that region of the field, and the heatmap represents the average amount of time this player has spent inside that region. This value is normalized by the maximum amount of time expended by the player in a specific region. Each row represents different instances of the game: the first row are all instances of the game (attacking and defending phases combined), the second is the attacking phase, and the third is the defending phase. In this case, the spatial partition is a combination of the ones proposed in Figs. 1,2, creating a 5x4 grid.


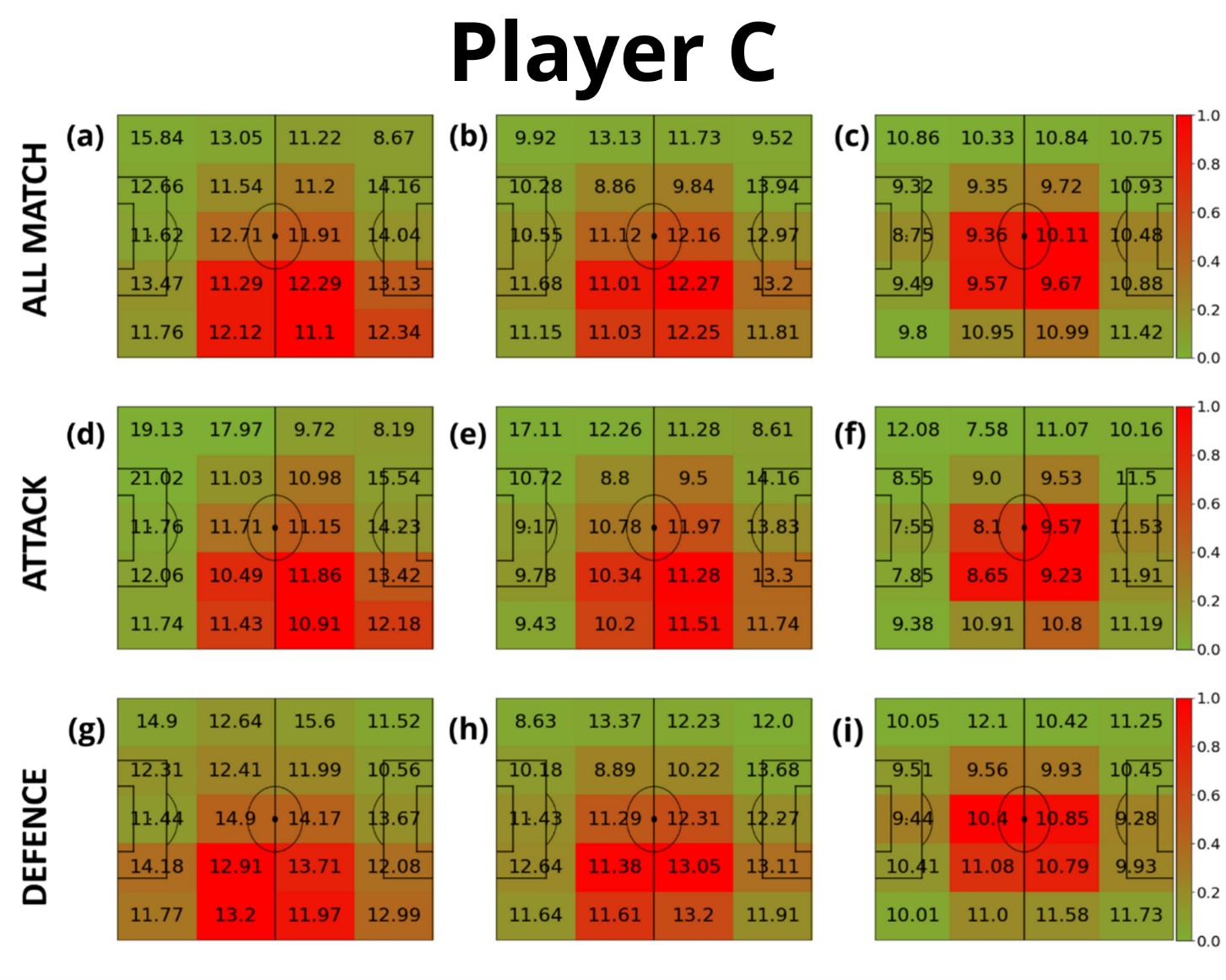
**Figure S6.** Mean velocity (in km/h) of Player C (midfielder) in terms of his distance to the ball. The intervals of the distance to the ball were: [0, 3) meters (first column), [3, 10) meters (second column) and (10, inf) meters (last column). Note that the values displayed in each zone is the average velocity of the player in that region of the field, and the heatmap represents the average amount of time this player has spent inside that region. This value is normalized by the maximum amount of time expended by the player in a specific region. Each row represents different instances of the game: the first row are all instances of the game (attacking and defending phases combined), the second is the attacking phase, and the third is the defending phase. In this case, the spatial partition is a combination of the ones proposed in Figs. 1,2, creating a 5x4 grid.


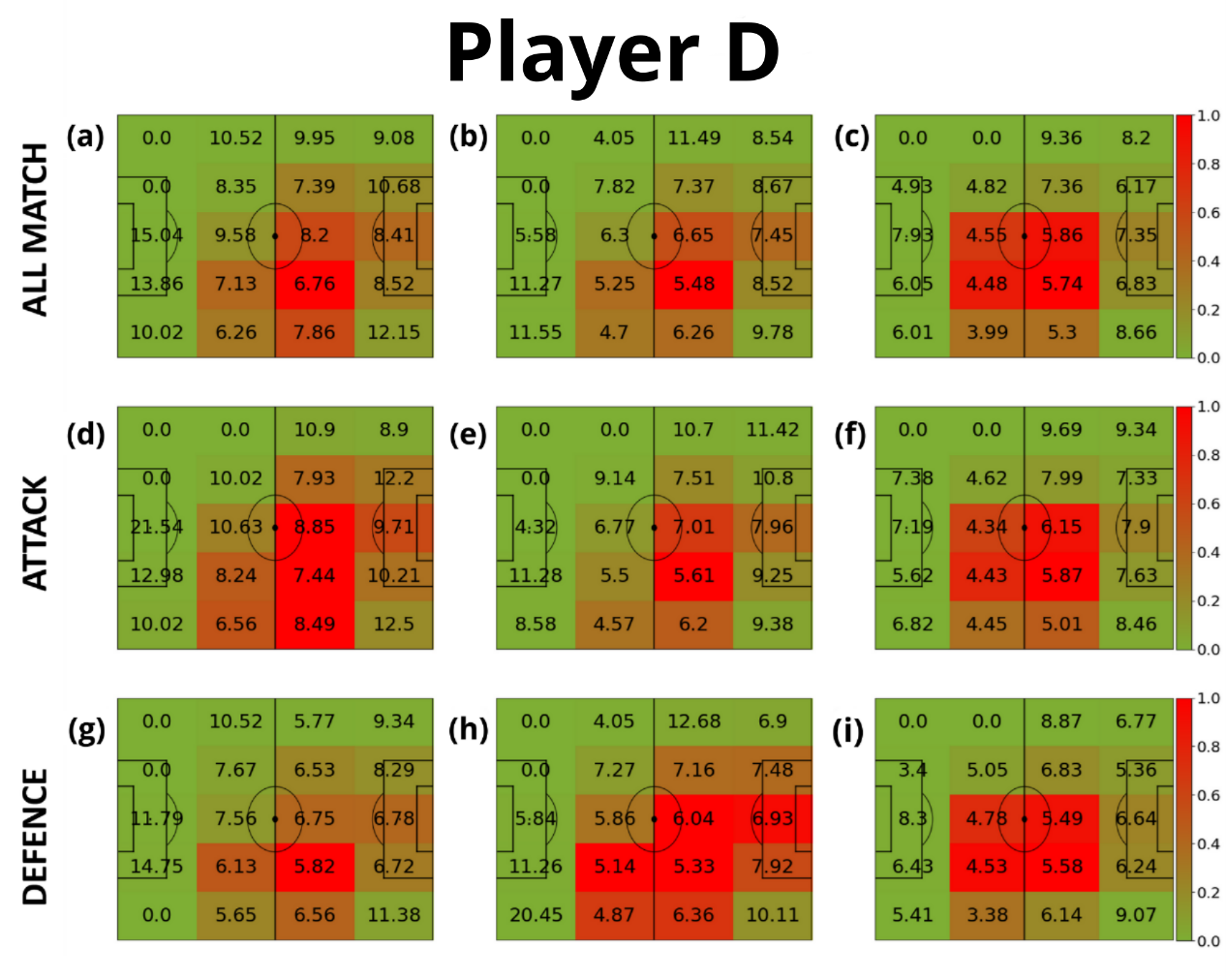


**Figure S7.** Mean velocity (in km/h) of Player D (forward) in terms of his distance to the ball. The intervals of the distance to the ball were: [0, 3) meters (first column), [3, 10) meters (second column) and (10, inf) meters (last column). Note that the values displayed in each zone is the average velocity of the player in that region of the field, and the heatmap represents the average amount of time this player has spent inside that region. This value is normalized by the maximum amount of time expended by the player in a specific region. Each row represents different instances of the game: the first row are all instances of the game (attacking and defending phases combined), the second is the attacking phase, and the third is the defending phase. In this case, the spatial partition is a combination of the ones proposed in Figs. 1,2, creating a 5x4 grid.
